# Plasticity of Adipose Tissue-Derived Stem Cells and Regulation of Angiogenesis

**DOI:** 10.3389/fphys.2018.01656

**Published:** 2018-11-26

**Authors:** Yulia A. Panina, Anton S. Yakimov, Yulia K. Komleva, Andrey V. Morgun, Olga L. Lopatina, Natalia A. Malinovskaya, Anton N. Shuvaev, Vladimir V. Salmin, Tatiana E. Taranushenko, Alla B. Salmina

**Affiliations:** ^1^Department of Biochemistry, Medical, Pharmaceutical and Toxicological Chemistry, Krasnoyarsk State Medical University named after Prof. V.F. Voino-Yasenetsky, Krasnoyarsk, Russia; ^2^Research Institute of Molecular Medicine and Pathobiochemistry, Krasnoyarsk State Medical University named after Prof. V.F. Voino-Yasenetsky, Krasnoyarsk, Russia; ^3^Department of Pediatrics, Krasnoyarsk State Medical University named after Prof. V.F. Voino-Yasenetsky, Krasnoyarsk, Russia; ^4^Department of Medical and Biological Physics, Krasnoyarsk State Medical University named after Prof. V.F. Voino-Yasenetsky, Krasnoyarsk, Russia

**Keywords:** adipose tissue, adipocyte, stem cell, endothelial cells, angiogenesis, vasculature-on-chip

## Abstract

Adipose tissue is recognized as an important organ with metabolic, regulatory, and plastic roles. Adipose tissue-derived stem cells (ASCs) with self-renewal properties localize in the stromal vascular fraction (SVF) being present in a vascular niche, thereby, contributing to local regulation of angiogenesis and vessel remodeling. In the past decades, ASCs have attracted much attention from biologists and bioengineers, particularly, because of their multilineage differentiation potential, strong proliferation, and migration abilities *in vitro* and high resistance to oxidative stress and senescence. Current data suggest that the SVF serves as an important source of endothelial progenitors, endothelial cells, and pericytes, thereby, contributing to vessel remodeling and growth. In addition, ASCs demonstrate intriguing metabolic and interlineage plasticity, which makes them good candidates for creating regenerative therapeutic protocols, *in vitro* tissue models and microphysiological systems, and tissue-on-chip devices for diagnostic and regeneration-supporting purposes. This review covers recent achievements in understanding the metabolic activity within the SVF niches (lactate and NAD+ metabolism), which is critical for maintaining the pool of ASCs, and discloses their pro-angiogenic potential, particularly, in the complex therapy of cardiovascular and cerebrovascular diseases.

## Adipose Tissue-Derived Stem Cells: Origin, Expression Patterns, and Niche Requirements

At present, adipose tissue is being recognized as an important organ with metabolic and regulatory roles and high regenerative potential. Adipose tissue responds to (patho)physiological stimuli such as fasting and meals (white adipose tissue) and hypothermia (brown adipose tissue) (Jokinen et al., 2017). The metabolic profile of mature adipocytes consists of reactions of lipogenesis, lipolysis, glycolysis, and oxidative phosphorylation and is controlled generally by the number and activity of mitochondria (Coelho et al., 2013).

Metabolically active brown adipose tissue is found as depots in newborns (in perivascular and periorgan visceral areas) as well as in adults (in cervical, supraclavicular, mediastinal, paravertebral, and suprarenal regions) (Silva et al., 2014). Moreover, adult perivascular adipose tissue (PVAT) remains the characteristic of both white and brown fat and its metabolic activity might be critical for controlling vascular tone, vessels remodeling, angiogenesis, and thermogenesis. Particularly, PVAT-derived adiponectin activates BKCa channels in smooth muscle cells (SMCs) resulting in vasodilation (Lynch et al., 2013). Aging-associated vascular dysfunction is coupled to aberrant browning of PVAT and local adenosine production (Kong et al., 2018), reduced PVAT NO production, and altered vasodilation as evident in the offspring of obese rats (Zaborska et al., 2016). Thus, it is not surprising that aberrant metabolic activity of adult PVAT results in deregulation of vasomotor mechanisms due to imbalanced production of vasoactive substances acting at neighboring endothelial and SMCs. It leads to the development of attenuated vasodilation as it was shown recently in aged spontaneous hypertensive rats (SHRs) with specific mitochondrial deficits. Particularly, Kong et al. (2018) found that there was significantly lowered expression of uncoupling protein 1 (UCP1) and peroxisome proliferator-activated receptor γ (PPARγ) in PVAT obtained from a 16-week-old SHR compared to an 8-week-old SHR, with the corresponding decline in the vasodilative effect and adenosine production in PVAT in the 16-week-old SHR. However, basal levels of UCP1 and PPARγ expression in PVAT of SHR were higher than in the control group, which was associated with smaller adipocyte size and lower lipid content (Kong et al., 2018).

Adipogenesis in the white adipose tissue requires proliferation and differentiation of adipose-derived mesenchymal stem cells (ADMSCs) that localize along blood capillaries (Fu et al., 2014). Adipose tissue-derived stem cells (ASCs) (ADMSCs) attract much attention from biologists and bioengineers, particularly because of the potential of ASCs to differentiate into a huge number of cells (i.e., cells of osteogenic, chondrogenic, white adipogenic, and brown adipogenic lineages, cardiomyocytes, cardiac pacemakerlike cells, SMCs, endothelial cells, neurons, etc.) (Zavan et al., 2010; Nagata et al., 2016; Sun et al., 2018). Attention should be paid to the observations that the clonogenic ability of ASCs seems to be reduced significantly in obesity and metabolic syndrome (Silva et al., 2015; Oliva-Olivera et al., 2017). Thus, the clonogenic potential of ASCs is affected by systemic and local factors, i.e., by microenvironmental alterations caused by deregulated metabolism in the adipose tissue or by systemic alterations caused by cytokines, toxins, and hormones.

In addition to ASCs/ADMSCs, other cell populations contribute to adipogenesis: adipose progenitors and preadipocytes. As it was summarized by Berry et al. (2015) from several *in vivo* studies, early adipocyte progenitors (APs) express CD24, CD29, CD34, Sca-1, and PDGFR2 and are negative in the expression of CD45 and CD31, whereas preadipocytes do not express CD24.

The ASCs with self-renewal properties localize in the stromal vascular fraction (SVF) being present in a vascular niche, demonstrate region-specific expression profile, and express mural cell markers such as platelet-derived growth factor receptor (PDGFRβ), neural/glial antigen NG2 as well as CD24 and PPARγ (Berry et al., 2013; Rezai Rad et al., 2017; Tran et al., 2018). Being cultured *in vitro* or assessed *in vivo*, ASCs display a spindle-shaped morphology, lack the intracellular lipid droplets in contrast to mature adipocytes, and express mesenchymal markers, i.e., CD90, CD105, CD73, CD44, and CD166 (Sullivan et al., 2015; Frese et al., 2016). Slight expression of other proteins, i.e., β-III-tubulin, VEGF, PDGFRβ, etc., was registered in ASCs (Zemelko et al., 2014; Mildmay-White and Khan, 2017). In sum, a recent systematic review revealed that the following markers are attributed to ASCs phenotype: CD90, CD44, CD29, CD105, CD13, CD73, CD166, CD10, CD49e, and CD59 (positive markers), while CD31, CD45, CD14, CD11b, CD34, CD19, CD56, and CD146 (negative markers) (Mildmay-White and Khan, 2017). However, attention should be paid while selecting an appropriate marker for studies in humans and rodents. As an example, mouse ASCs have been found to be positive for mesenchymal markers CD90 and CD105, Nanog, SSEA-1, CD106, and VEGFR-1 and negative for hematopoietic markers CD34 and CD45 (Luna et al., 2014), whereas human ASCs may demonstrate quite a different expression of CD90 and CD105, which correlates with their differentiation potential (Baer, 2014). The situation is complicated further by the different levels of expression of markers in ASCs isolated from various regions, i.e., from subcutaneous and visceral fat (Ong et al., 2014).

White and brown ASCs are different in the origin and lineage characteristics, particularly, white adipose stem cells originate from Myf5 (myogenic regulatory factor) negative progenitors, whereas brown adipose stem cells originate from myogenic lineage and express Myf5 (Algire et al., 2013). In general, adipose progenitors develop in close relation to vasculature; express PPARγ, stem cell antigen-1 (SCA-1), CD34, smooth-muscle actin (SMA), PDGFRβ, and chondroitin sulfate proteoglycan 4 (NG2), and VE-cadherin; and may, probably, have endothelial (but not hematopoietic) origin (Algire et al., 2013; Onogi et al., 2017). As an example, the expression profile of perivascular ASCs contains (in addition to mesenchymal markers) some markers of endothelial cells (i.e., platelet endothelial cell adhesion molecule CD31/PECAM-1, vascular cell adhesion molecule CD106/VCAM-1, melanoma cell adhesion molecule CD146/MCAM, and activated leukocyte cell adhesion molecule CD166/ALCAM) and perivascular cells (i.e., 3G5 ganglioside expressed in pericytes) (Zannettino et al., 2008). Presumably, such controversies in results of assessment of expression patterns in ASCs could be caused by different protocols applied for cell purification and characterization.

Some authors suggest that both endothelial cells and pericytes are precursors of adipose progenitors as confirmed by the scrupulous VE-cadherin promoter-driven lineage-tracing experiments in rodents and experiments with cultured human adipose tissue: endothelial cells of developing white and brown adipose tissue capillaries are a source of adipocyte precursors; thereby, adipocytes and endothelial cells are plastic enough to undergo interconversion (Tran et al., 2012).

Perivascular location of ASCs might suggest their contribution to a well-known endocrine and paracrine activity of adipose tissue surrounding medium- and large-sized vessels in the context of the so-called adipose-vascular coupling (Gollasch and Dubrovska, 2004; Gollasch, 2017; Gollasch et al., 2018). The PVAT is in direct contact with the vessel adventitia, which is considered as a progenitor cell niche within the vessel wall inhabited by a huge number of stem cells and progenitors, i.e., endothelial progenitor cells (EPCs), progenitors for SMCs, mesenchymal stem cells (MSCs), mesangial cells coexpressing both endothelial and myogenic markers, and organ- and tissue-specific progenitors, i.e., neural stem cells in neural tissue (Ergun et al., 2011; Majesky et al., 2011). Thus, it is not surprising that ASCs reside in this particular area as a part of the MSCs population (adventitial vasculogenic zone). It is important to note that PVAT (particularly in the thoracic, but not in the abdominal area) are originated from Myf5- precursors and may not share myogenic characteristics typical for “classic” brown ASCs (Hildebrand et al., 2018).

The ASCs with multilineage differentiation potential have been isolated successfully from metabolically active brown adipose tissue (Silva et al., 2014). In contrast to white ASCs, visceral (i.e., mediastinal) brown adipose tissue-derived ASCs (BADSCs) demonstrate the highest expression of transmembrane protein 26 (TMEM26) and CD137 that also can be considered as markers of beige adipose tissue (white adipose tissue is enriched in brown UCP1-expressing thermogenically competent adipocytes) (Silva et al., 2014). Therefore, BADSCs might be defined as a population of stem cells with promising applications either in tissue engineering or in therapeutic protocols (Yang et al., 2017; Chen et al., 2018).

The clonogenic activity of ASCs is supported by the microenvironment established within the SVF by fibroblasts, mature brown adipocytes and preadipocytes, pericytes, immune cells (macrophages, lymphocytes), and endothelial cells/EPCs (Chazenbalk et al., 2011; Kim et al., 2014). Strictly speaking, adipogenic niches are required for adipogenesis, i.e., in the conditions of brown adipogenesis induction (beta-adrenergic stimulation, hypothermia) or in white adipogenesis induction (high-fat feeding) (Lee et al., 2012, 2013; Lee Y.H. et al., 2015). It is interesting that stimulation of adipogenesis is associated usually with remodeling of the niche-resembling establishment of the pro-inflammatory microenvironment (activation of local macrophages) (Lee et al., 2013).

Establishment of the clonogenic niche in the perivascular adipose fraction provides a microenvironment enriched with factors contributing to the regulation of stemness and self-renewal of ASCs. As an example, stimulation of PPARγ in ASCs results in activation of PDGFRβ and VEGFR, further leading to extensive local vascular sprouting and increased vessel niche affinity for ASCs (Jiang et al., 2017). Thus, the local concentration of PPARγ ligands (fatty acids derivatives including nitroalkene fatty acids) might be one of the critical factors for controlling the occupancy of ASCs within the perivascular niche. Expression of CD73 in ASCs suggests involvement of adenosine-mediated mechanisms in the regulation of behavior of stem cells. In this scenario, CD39 expressed by stromal cells or SVF regulatory T-cells may produce adenosine monophosphate (AMP) from adenosine triphosphate (ATP), whereas CD73 expressed by ASCs can convert AMP to adenosine (de Oliveira Bravo et al., 2016; Donninelli et al., 2017). Presence of CD203+ macrophages in the adipose tissue and, particularly, in the SVF niche (Faris et al., 2012; Silva et al., 2017) leads to the proposal of an alternative mechanism. Particularly, adenosine might be produced in the SVF niche due to conversion of NAD+ into ADP-ribose by means of the activity of CD38/NAD+-glycohydrolase expressed in stem cells, followed by ADP-ribose metabolism to AMP due to activity of CD203a expressed by macrophages. Then, the CD73-mediated reaction might result in adenosine production as it was proposed before for activated immune cells (Horenstein et al., 2013). Regardless of the way of production, adenosine is a well-known regulator of development of stem cells (Carroll et al., 2012; Jing et al., 2015). Mesenchymal stem cells as well as adipocytes in white and brown adipose tissue express adenosine receptors (Gharibi et al., 2011; Tozzi and Novak, 2017); therefore, local production of adenosine may control the development of ASCs. Therefore, it is very reasonable that modulation of adenosine receptors regulates adipogenesis: adenosine activates brown adipose tissue adipocytes and brown like beige adipocytes (Gnad et al., 2014), promotes differentiation, and blocks lipolysis in adipocytes (Eisenstein and Ravid, 2014).

Adipogenesis and production of adipose-derived regulatory factors are compromised in chronic vascular dementia, neurodegeneration, and cardiovascular diseases (Zhou and Qin, 2012; Ishii and Iadecola, 2016). Adipose-derived factors contribute to progression of vascular dementia and Alzheimer’s disease (Ishii and Iadecola, 2016). High salt intake provokes adipogenesis and local inflammation (Park et al., 2017). Aberrant epicardial adipogenesis contributes to pathogenesis of cardiac arrhythmias (Yamaguchi et al., 2015) with the distribution of adipose tissue being changed. Also, there is a negative correlation between adipose tissue-produced leptin levels and amyloid-beta concentrations in cerebrospinal fluid in females with Alzheimer’s disease (Diehl-Wiesenecker et al., 2015). Thus, it is very reasonable that adipose stem cells or their derivatives have been tested in humans and animal models as a regenerating therapeutic tool. However, it is too early to conclude on promising results in preclinical animal studies or clinical trials for myocardial infarction (Lee H.W. et al., 2015; Kim et al., 2016; Bobi et al., 2017; Joo et al., 2017), ischemic cardiomyopathy (Suzuki et al., 2015), stroke (Gutiérrez-Fernández et al., 2013; Grudzenski et al., 2017), and Alzheimer’s disease (Lee et al., 2018). In light of these introductory remarks, it is apparent that deciphering the novel regulatory mechanisms of the self-renewal and differentiation of ASCs would promote application of advanced and safe protocols for regenerating therapy. The next chapters will cover some recent achievements in understanding the metabolic activity within the SVF niches, which is critical for maintaining the pool of ASCs, and disclosures of their regenerative, particularly, pro-angiogenic potential.

## Metabolism of Lactate and NAD+ in Adipose Clonogenic Niches

Mobilization of fatty acids from adipocytes and production of lactate serve as a major mechanism of energy supply, and uncoupling of oxidation and phosphorylation in adipocyte mitochondria supports thermogenesis (Berry et al., 2013). Recently, it has become clear that glycolytic production of lactate and its transport via monocarboxylate transporters (MCTs) control metabolic activity of brown fat cells underlying thermogenesis. Prolonged optogenetic activation of ChR2-expressing sympathetic neurons innervating brown adipose tissue resulted in thermogenic responses and extensive GLUT1-mediated glucose uptake needed for lactate production and MCT1-mediated lactate transport and its utilization as an energy substrate (Jeong et al., 2018).

How do such metabolic and regulatory activities of the adipose tissue affect the behavior of ASCs? The ASCs, as all other cells with multilineage differentiation characteristics, greatly depend on glycolysis whose metabolites are required for maintaining the pool of stem cells and preventing their inappropriate (uncontrolled) recruitment (Burgess et al., 2014). Stem cells themselves as well as neighboring cells serve as a source of lactate. Glycolysis in adipose cells is required absolutely for their functional activity and differs in the adipose tissue obtained from various anatomical locations (Ferng et al., 2016). Glycolytic activity of brown fat cells depends on transcriptional activation of HIF-1, expression of glucose transporters (GLUT) expression, and glycolytic enzymes (Basse et al., 2017). As an example, activation of PDGFRβ is required for effective glucose uptake in the adipose tissue (Onogi et al., 2017). It was observed that adipogenic induction is associated usually with transition from glycolytic mechanism to mitochondrial respiration (Drehmer et al., 2016; Zheng et al., 2018).

Proteomic studies reveal that expression of glycolytic enzymes in mature adipocytes and in SVF cells is different (Kheterpal et al., 2011), thereby, suggesting that the niche establishment is associated with specific changes in energy production in the surrounding cells of ASCs. As it was shown in other clonogenic niches located in bone marrow, brain, or in tumor loci, elevated production of lactate is a prerequisite for maintaining stem cells pool, whereas changes in lactate concentrations serve as a signal for cell proliferation and differentiation being responsible for the so-called Warburg’s effect and reverse Warburg’s effect (increased glucose uptake followed by extensive glycolysis in stem/progenitor cells or in surrounding accessory cells, respectively) (Álvarez et al., 2014; Malinovskaya et al., 2016). Along the process of differentiation, preadipocytes acquire a glycolysis-independent mode of energy production and come to utilize more fatty acids (Roberts et al., 2009). As expected, expression of lactate transporters – MCT1 and MCT4 – is progressively elevated in adipocytes during white and brown adipogenesis, which is important for lactate influx and support of mitochondrial energy production (Petersen et al., 2017). Lactate serves as a “browning” signal for white adipose cells: in an MCT-dependent manner, it increases the expression of UCP1 and mitochondrial activity (Vergnes and Reue, 2014). Moreover, brown adipose cells most abundantly express GPR81 as lactate receptor (Liu et al., 2009), and in mature adipocytes, activation of GPR81 receptors leads to the inhibition of lipolysis (i.e., in insulin action) (Rooney and Trayhurn, 2011). It is especially interesting to note that expression of GPR81 is required for stem cell maintenance (Choi et al., 2015), but whether BADSCs express and use GPR81 activity for their own needs within the SVF niche remain to be evaluated.

In white adipose tissues, expression of glycolytic enzymes is elevated during growth of adipocytes and glycolysis is activated in mitochondrial uncoupling (Sabater et al., 2014). It should be noted that brown adipocytes differ from white adipocytes in that brown adipocytes convert a greater proportion of metabolized glucose into (lactate + pyruvate) and a smaller proportion into fatty acids (*de novo* lipogenesis) than do white adipocytes (Saggerson et al., 1988). Since reduction of *de novo* lipogenesis may correspond to the insulin-resistant state in adipose tissue or may reflect secondary changes in processes requiring fatty acids-derived products as regulatory factors (protein acetylation, PPARγ signaling) (Guilherme et al., 2017), one can assume that suppression of glycolytic production of lactate in differentiating SVF adipocytes should relate to differentiation-coupled changes in epigenetic mechanisms. Adult cow BADSCs cultured *in vitro* demonstrate reduction of histone H3K9 acetylation (marker of transcriptionally active chromatin), presumably, due to reduced pluripotency potential of the stem cells and their commitment to a particular lineage or to cellular senescence (Abouhamzeh et al., 2015), which is a general phenomenon in the differentiation of stem cells. Thus, dynamic changes in the glycolytic activity of BADSCs as well as surrounding cells within the adipose niches would affect differentiation of stem cells due to secondary alterations in lactate bioavailability and fatty acids metabolism.

Hydrogen sulfide (H_2_S) serves as a regulator of glycolysis in several cell types (Lee et al., 2014). The PVAT is an important source of endogenously produced H_2_S (Schleifenbaum et al., 2010; Gollasch, 2017). On the contrary, H_2_S supports the proliferation and viability of ASCs (Dongó et al., 2014; Aykan et al., 2015); therefore, one can assume that it might relate to H_2_S-mediated effects on glycolytic production of lactate within the SVF niche. This possible link between H_2_S-producing ability and glycolysis efficacy in SVF cells remains to be evaluated.

Another important property of glycolysis and lactate production is intracellular NAD+ regeneration coupled to pyruvate-lactate conversion. Therefore, greater glycolysis and higher lactate production in the early stages of the development of the BADSCs could be important for maintaining intracellular NAD+ levels adequate for the actual metabolic needs of actively proliferating and differentiating cells. However, this is true only for conditions when mitochondrial respiration is suppressed because action of lactate as mitochondrial fuel would require reverse conversion and rise in NADH concentrations. Thus, differentiation of cells within the SVF niche should be associated with depletion of the intracellular pool of NAD+.

Adipose cells are well equipped with NAD+-generating and -consuming machinery. As an example, expression of visfatin (nicotinamide phosphoribosyltransferase involved in the salvage pathway of NAD+ biosynthesis) is prevalent in visceral adipose tissue (Coelho et al., 2013). The NAD+-consuming enzymes are NAD+-glycohydrolases (CD38, CD157), poly (ADP-ribose) polymerase, and histone deacetylases (HDAC or sirtuins). The NAD+-glycohydrolase/CD38 is a receptor and an enzyme-generating second messenger with Ca2+-mobilizing activity (i.e., cyclic ADP-ribose) and is expressed widely in brain, liver, adipose tissue, and immune cells. Activity of CD38 regulates many biological functions, i.e., immune response, insulin secretion, contraction of cardiomyocytes, glial activation, and secretion of neuropeptides (Malavasi et al., 2008; Salmina et al., 2009; Lopatina et al., 2012). A progressive increase in the expression of CD38 in adult adipose tissue in aging relates to depletion of intracellular NAD+ levels and can be responsible for mitochondrial dysfunction in a sirtuin-dependent manner (Camacho-Pereira et al., 2016). Recently, a complex experimental approach with flow cytometry, cell-sorting protocols, and immunohistochemistry revealed that CD38 should be considered as a marker of preadipocytes that are committed to the adipogenic differentiation program (CD45- CD31- CD34-low CD38+ cells); therefore, the number of CD38-immunopositive cells with reduced proliferative potential is increased in extensive adipogenesis (Carriere et al., 2017). In support of this observation, CD38 deficiency was found to inhibit adipogenesis-activating fatty acid synthase via the Sirt1/PPARγ-signaling pathway (Wang et al., 2018). It is tempting to speculate that an increase in CD38 expression might occur within the SVF niche at the stage of differentiation of preadipocytes and correspond to relative suppression of glycolysis. As a result of the two mechanisms (overexpression of CD38 and reduction of glycolytic flux), intracellular NAD+ levels in ASCs may decrease dramatically.

**FIGURE 1:**
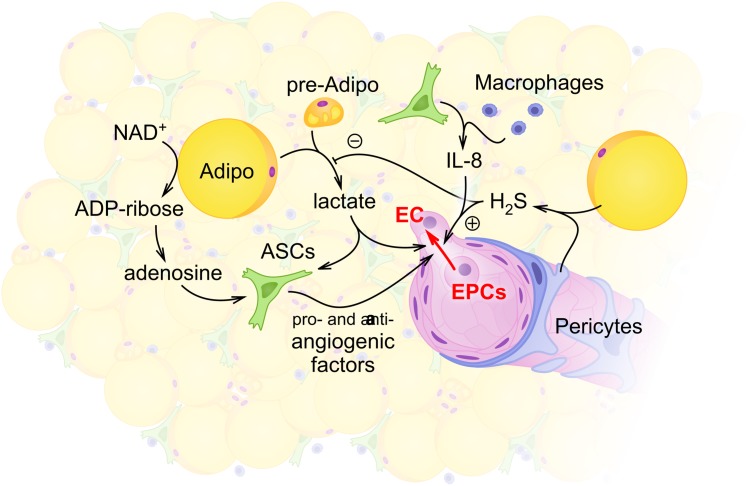
Schematic illustration of intercellular communications within the stromal vascular fraction (SVF) with the focus on adipocyte- and ASCs-mediated regulation of angiogenesis. Within the SVF, adipocytes (Adipo) and preadipocytes (pre-Adipo) serve as a major source of glycolytically produced lactate. Elevated levels of lactate in the extracellular space support proangiogenic activity of ASCs whose maintenance also depends on glycolytic flux and mitochondrial respiration. Some other locally produced molecules [hydrogen sulfide H_2_S in pericytes (P) or adipocytes; adenosine and interleukin-8 (IL-8) in ASCs or macrophages] contribute to angiogenesis control within the SVF. As a result, activity of endothelial cells (ECs) and EPCs provides angiogenesis and vascular remodeling adjusted to the current metabolic and functional needs of adipose tissue.

It is apparent that such changes in the production and utilization of lactate may correspond also to the activity of HDACs; sirtuins. As it was suggested, energy shortage under the conditions of caloric restriction, starvation, and exercise activate sirtuins, whereas high-fat feeding suppresses the activity of sirtuins (Jokinen et al., 2017). Expression of sirtuins in adipose stem cells varies in a region-specific manner (Mariani et al., 2017). In the brown adipose tissue, the activity of various sirtuins is required for the regulation of mitochondrial number, mitochondrial respiration, glucose uptake, and thermogenesis, whereas, in the white adipose tissue, other effects of sirtuins activity have been detected (control of adipokines production, adipogenesis) (Jokinen et al., 2017). Particularly, sirtuins 1 and 2 suppress adipogenesis under fasting conditions (Rodriguez et al., 2013), whereas mitochondrial sirtuin 3 in brown adipocytes affects positively the expression of UCP1 and thermogenesis (Ansari et al., 2017). Thus, NAD+ bioavailability may dictate the expression levels and activity of sirtuins in ASCs and neighboring cells [i.e. (pre)adipocytes], thereby, providing a mechanistic link between the intensity of glycolysis, metabolism, and clonogenic capacity of ASCs. Figure 1 illustrates the role of lactate and NAD+ metabolism in supporting the functional competence of ASCs.

## Pro-Angiogenic Activity Within Adipose Clonogenic Niches

Reciprocal interactions connect two mechanisms – adipogenesis and angiogenesis. Expansion of adipose tissue requires capillary growth; therefore, either in embryonic or in adult stages of ontogenesis, the development of adipose tissue corresponds to the intensity of local angiogenesis/vasculogenesis.

The SVF niches serve as a platform for angiogenesis and neovascularization, where VEGF and PDGF appear as major regulatory signals (Klar et al., 2016; Bora and Majumdar, 2017). Current data suggest that SVF serves as an important source of endothelial progenitors, endothelial cells, and pericytes, thereby, contributing to vessel remodeling and growth (Han et al., 2015). Adipose tissue itself is a big source of angiogenesis activators and inhibitors (Cao, 2007). Pro-angiogenic activity of PVAT is provided by the secretion of soluble factors with strong angiogenic potential, i.e., vascular endothelial growth factor (VEGF), acidic fibroblast growth factor (aFGF), monocyte chemoattractant protein-1 (MCP-1), insulin like growth factor-binding protein-3 (IGFBP-3), glia-derived neurotrophic factor (GDNF), and hepatocyte growth factor (HGF) (Voros et al., 2005; Rittig et al., 2012; Horimatsu et al., 2017). The CD248-immunopositive SVF ASCs show higher pro-angiogenic potential than do CD248-cells (Zielins et al., 2015; Brett et al., 2017). The CD248 (endosialin) is a positive regulator of the development of pericytes (Tomkowicz et al., 2010). In mice, endothelial cells in vessels within the adipose tissue may give rise to new adipocytes coupled via tight junction proteins at the very early stages of their differentiation *in vivo* (Tran et al., 2012). Pro-angiogenic activity of adipose niche cells has been confirmed numerous times *in vitro*: 14 days coculture of mice endothelial cells and ASCs drives Wnt-regulated angiogenesis and functional vessel formation in 3D collagen matrices (Cai et al., 2017), ASCs support greater sprouting of endothelial cells being cocultured on bioengineered polycaprolactone/gelatin nanofibrous scaffolds (Kook et al., 2018), and coculture of ASCs with adipose-derived microvascular endothelial cells results in enhanced vascular network formation during 14 days of incubation period *in vitro* (Freiman et al., 2016).

Antiangiogenic activity of adipose tissue relies on the stimulated secretion of adiponectin, endostatin, thrombospondin-1, soluble VEGF receptor 2 type, and transforming growth factor-beta (TGFβ) (Cao, 2007). The functional activity of adipocytes controls secretion of pro- and antiangiogenic factors, i.e., cold stress stimulates production of VEGFA in brown adipose tissue and it directly activates proliferation of adipocytes precursor and endothelial cells (Wang et al., 2015). On the contrary, fatty acids mobilized from adipocytes could serve as ligands for PPAR receptors, thereby, affecting endothelial cells, i.e., as it was shown for PPARα receptors expressed in brain microvascular endothelial cells (BMECs) within the blood-brain barrier (BBB), thus leading to hyperpermeability of BBB (Caroline et al., 2009). Exosomes released from ASCs and containing microRNA-181b-5p are able to stimulate angiogenesis through activation of BMECs, particularly, under hypoxic conditions (Yang et al., 2018).

As it was mentioned earlier, endothelial cells and pericytes may serve as precursors of ASCs (Tran et al., 2012). Indeed, SVF contains a CD31-, S100+ cell type that can differentiate into adipocytes and endothelial cells and CD31+ SVF cells can be converted to adipocytes *in vitro* (Wosnitza et al., 2007). Human SVF-derived cells retrieved from the adipose tissue can differentiate into endothelial cells, and being transplanted into mice are able to support angiogenesis *in vivo*, whereas dedifferentiated mature human adipocytes display the potential to acquire the endothelial phenotype *in vitro* and promote vessel-like tube formation (Planat-Benard et al., 2004). Recent data suggest that bone marrow-derived cells (probably mesenchymal cells and endothelial progenitors) may also contribute to the pool of adipocytes (Arner and Rydén, 2017); even some controversial observations exist (Koh et al., 2007) that might be explained by the presence of distinct subpopulations of progenitors (Jiang et al., 2014).

Contacts between endothelial cells and ASCs are necessary for inducing pro- or antiangiogenic secretory activity in stem cells. As an example, endothelial cells contacting ASCs *in vitro* induce expression of activin A, which is an important factor in controlling vasculogenesis (Merfeld-Clauss et al., 2015). Assessment of vasculogenesis *in vitro* in 3D fibrin matrices demonstrated that direct interactions of human umbilical vein endothelial cells (HUVECs) and ASCs are required for promotion of angiogenesis: culture of HUVEC alone due to extensive secretion of VEGF, angiogenin, angiopoietin-1, MCP-1, matrix metaloproteinases, and many other proangiogenic factors as well as angiogenesis inhibitors (i.e., thrombospondin-1, platelet factor 4, serpin F1, etc.) (Rohringer et al., 2014). The most interesting finding of this study was a mechanism of acquiring pericyte phenotype by ASCs cocultured with HUVEC, especially in the regions of newly formed tubes bifurcations within the vascular network. It might be pertinent to note that human pericytes obtained from adipose tissue display better differentiating phenotypes compared to pericytes developed from MSCs (Pierantozzi et al., 2015).

Endothelial progenitor cells (EPCs) with high angiogenic potential can be isolated easily from SVF (Van Pham et al., 2016) and used further for bioengineering purposes. It is important to mention that adipose tissue-derived EPCs demonstrate higher proliferative potential than EPCs obtained from ASCs themselves (Zhou et al., 2015). The EPCs isolated from adipose tissue have been shown also to be present in the microvessel fraction, particularly, in sub- or periendothelial space close to pericytes (Ergun et al., 2011). Taking into consideration the close relations of adipocytes and endothelial cells as well as the high degree of their interlineage plasticity, mesenchymal-to-endothelial transformation of ASCs (Zhou et al., 2015), one may assume that this phenomenon contributes to various (patho)physiological processes as was shown earlier for several types of vessel remodeling and neovascularization (Ubil et al., 2014). Since these events are driven usually by local chronic hypoxia, initiation of mesenchymal-to-endothelial transition of ASCs should be associated with excessive adipogenesis and enlargement of adipose tissue clusters. It is well known that hypoxia is associated always with lactate accumulation; therefore, local production of lactate within SVF could regulate differentiation of ASCs into endothelial cells. Indeed, lactate has been shown to serve as a positive regulator of angiogenesis, including model systems with specially designed matrices (lactate-releasing or demonstrating gradients in lactate concentrations) (Hunt et al., 2007; Porporato et al., 2012; Malinovskaya et al., 2016; Salmin et al., 2017), and lactate-enriched microenvironment within clonogenic niches might be able to promote vasculogenesis in the loci of extensive adipogenesis. The proangiogenic activity of lactate often requires IL-8-driven proliferation of endothelial cells (Polet and Feron, 2013), and adipose cells appear to be good producers of IL-8 into the systemic circulation (Bruun et al., 2004). Recent experimental data on the stimulated microgravity-induced angiogenesis-supporting expression pattern in ASCs (elevated levels of Serpin E1, Serpin F1, insulin growth factor binding protein (IGFBP), VEGF, and IL-8 in ASCs) (Ratushnyy et al., 2018) confirm partially the mechanism of lactate-mediated proangiogenic effects within the SVF niche.

In sum, the population of ASCs in SVF consists of pluripotent stem cells that could be considered as vasculogenic precursors able to differentiate into endothelial cells and pericytes to support angiogenesis/vasculogenesis and vessel remodeling. Thus, it is very reasonable to expect that ASCs-derived cells are very promising materials for establishment of bioengineered constructs for regenerative and vessel-replacement needs.

## Conclusion and Perspectives: Novel Vascular Engineering Strategies in Cardiovascular and Cerebrovascular Diseases

Initial attempts in utilizing the regenerative potential of ASCs have been connected with infusions of ASCs. Intracoronary infusion of ASCs in experimental animals with a model of transmural myocardial infarction resulted in increased left ventricle ejection fraction, elevated thickness of ventricular wall in the infarction area, and improved vascular density at the border zone at 4 weeks after the infusion (Valina et al., 2007). Systemic delivery of ASCs was applied in animal models of myocardial infarction (Hong et al., 2014). In parallel, application of ASCs has been progressing as target differentiation of ASCs *in vitro* to produce desirable cell lines, i.e., into beating cardiomyocytes due to epigenetic modifications of human ASCs or their coculture with rodent cardiac cells (Choi et al., 2010) and cardiac pacemakerlike cells due to transfection of rodent ASCs with TBX18 gene and their coculture with neonatal rodent cardiomyocytes (Yang et al., 2016).

Positive results have been obtained in rats with experimental ischemic stroke after intravenous administration of adipose tissue-derived MSCs (improvement of neurogenesis, oligodendrogenesis, synaptogenesis, and cerebral angiogenesis) (Gutiérrez-Fernández et al., 2013). In neonatal rats with perinatal hypoxic-ischemic brain injury, when ASCs were implanted with adipose stem cells-derived EPCs and neural progenitors, they demonstrated improved status (Hsueh et al., 2015). Alzheimer’s disease is known to have a significant vascular component in its pathogenesis (Kester et al., 2014); therefore, utilization of the proangiogenic capacity of ASCs might have therapeutic potential. However, various target processes seem to be involved in the positive action of ASCs and their derivates in experimental neurodegeneration. Neurogenic differentiation of ASCs could be achieved *in vitro* (Safford et al., 2002), while stimulation of endogenous neurogenesis was registered in mice with an Alzheimer’s disease model treated with ASCs (Yan et al., 2014). When human ASCs were injected intravenously or intracerebroventricularly into aging mice, improvements of their locomotor activity and cognitive function have been registered (Park et al., 2013). It is interesting that breakdown of the BBB seen in Alzheimer’s disease may contribute positively to the efficacy of ASCs-based therapy: intravenously injected human ASCs were able to reach brain tissue in transgenic Alzheimer’s disease model mice, but not in control mice (Ha et al., 2014). Another original approach was suggested to correct amyloid-beta proteolysis in the brain by means of neprilysin-carrying human ASCs-derived exosomes (Katsuda et al., 2015). This approach has been developed when neprilysin-enriched exosomes were found as products of activated ASCs (Katsuda et al., 2013). Thus, not only ASCs themselves of ASCs-originated neuronal cells, but also ASCs-derived exosomes could be considered as a therapeutic tool in Alzheimer’s type of neurodegeneration. Very recent data suggest that activated ASCs might be also efficient in the mouse model of Parkinson’s disease (Chi et al., 2018).

However, more preclinical studies and clinical trials are required since assessment of safety should be considered always in order to get rational conclusions on the clinical applications of ASCs in cardiovascular and cerebrovascular/neurodegenerative diseases (Toyserkani et al., 2017).

The next phase in the application of ASCs is linked to engineering the blood microvascular *in vitro* networks as a platform for drug testing or as a prototype for tissue implants based on ASCs cultured alone or with endothelial cells. Such attempts already have produced rather promising results (Murohara, 2009; Knezevic et al., 2017). Multilineage properties (assessed by differentiation of ASCs into a wide spectrum of cells) and high regenerative capacity (confirmed by analysis of angiogenic activity, senescence-resistant phenotype, and susceptibility to oxidative stress *in vitro*) of ASCs compared with bone marrow-derived stromal mesenchymal cells make them attractive candidates for bioengineering tasks. Particularly, their application on 3D scaffolds or in microfluidic systems, where regenerative potential of seeded cells or their response to the matrix architecture are of great importance, is very promising (Lee et al., 2016). Behavior of ASCs in microfluidic devices (strong proliferation and migration abilities) (Wadhawan et al., 2012) confirms good potential for the application of ASCs in dynamic cell models, multicellular ensembles, and microphysiological systems as well as for tissue-printing purposes (Zhang et al., 2017). As an example, the multi chamber dynamic system was developed successfully to study effects of various stimuli on ASCs differentiation toward myocardial phenotype (Pavesi et al., 2014).

Finally, ASCs could be considered as a source of cells for bioengineering constructions mimicking clonogenic niches *in vitro*. Establishment of a clonogenic/angiogenic microenvironment is a very complex problem whose solving would provide great progress in constructing implantable regeneration-supporting devices or supporting survival of grafted cells *in vivo*. Several attempts have been made to produce human SVF *in vitro* by placing ASCs and ASCs-supporting cells (pericytes, endothelial cells) on perfused scaffolds (Scherberich et al., 2010; Cerino et al., 2017; Costa et al., 2017). The results confirmed strong angiogenic potential of ASCs and release of proangiogenic factors and development of vascular network (Cerino et al., 2017; Costa et al., 2017). Recently, tissue-engineered vascular grafts with BADSCs have been proposed as promising novel alternatives to replace diseased vessels in cardiovascular and cerebrovascular diseases (Wang et al., 2016, 2017). Another task is very close on technological issues: development of ASCs-based adipose tissue microchip (“fat-on-chip”) with possible diagnostic, therapeutic, pharmacological, and toxicological applications (Loskill et al., 2016, 2017; Chen et al., 2017; Tanataweethum et al., 2017; Li and Easley, 2018).

**FIGURE 2:**
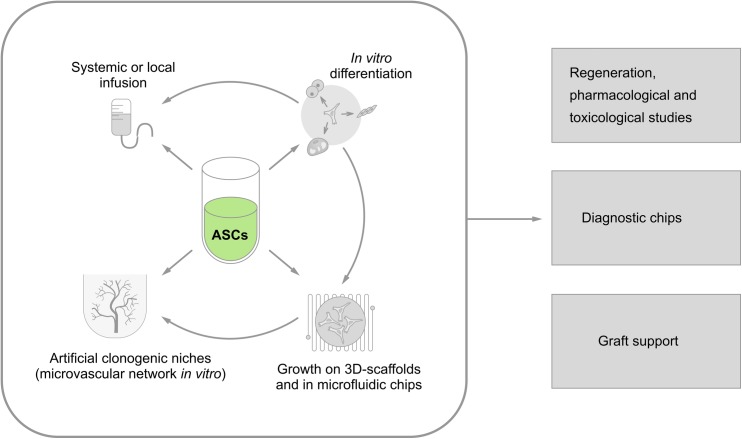
Current approaches to vascular engineering strategies utilizing ASCs as a source of vascular and perivascular cells. Isolated ASCs might be used for: (i) systemic (intravenous) or local (i.e., intracoronary) administration; (ii) *in vitro* differentiation toward the desired phenotype (myocardial cells, pacemakerlike cells, endothelial cells, smooth muscle cells, neuronal cells, etc.); (iii) establishment of 3D models and microfluidic systems *in vitro*; and (iv) development of artificial clonogenic niches for controlled *in vitro* production of stem cells supported by microvascular network). Then, all these approaches could be used in: (i) regeneration therapeutic protocols aimed to re-establish tissue components, including (micro)vessels; (ii) drug/xenobiotic testing *in vitro*; (iii) diagnostic devices in “lab-on-chip format”; (iv) supporting devices for grafted cells (as vascular scaffolds) or bioreactors for efficient generation of stem and progenitor cells *in vitro*.

All the earlier-mentioned approaches for the application of ASCs for diagnostic and therapeutic purposes are summarized in Figure 2.

In sum, remarkable progress has been made in the last decades in deciphering the molecular mechanisms of the functional activity of ASCs, particularly, in relation to their control of angiogenesis. Vascular scaffolds existing in real niches (i.e., in adipose tissue SVF, bone marrow niche, brain neurogenic niches, oligovascular niches) can be reproduced *in vitro*. Thus, adequate support for maintenance of stem cells and control of their differentiation suggests new approaches for the optimization of tissue-regeneration protocols, particularly, in the complex therapy of cardiovascular and cerebrovascular diseases.

## Author Contributions

YP, NM, TT, VS, and ABS conceived and wrote the manuscript. YK, OL, AM, AY, and ANS wrote the manuscript. AY designed the figures. All authors contributed to the final version of the manuscript. ABS supervised the project.

## Conflict of Interest Statement

The authors declare that the research was conducted in the absence of any commercial or financial relationships that could be construed as a potential conflict of interest.
